# Teaching Nutrition in Psychiatric Practice: Implementation of a Curriculum for Psychiatry Residents

**DOI:** 10.1007/s40596-025-02249-w

**Published:** 2025-10-30

**Authors:** Marta Karolina Mudd, Cara Angelotta

**Affiliations:** Northwestern University Feinberg School of Medicine, Chicago, IL USA

Mental health disorders, particularly depression and anxiety, are increasingly intertwined with physical health issues, such as diabetes, cardiovascular disorders, and select cancers, with diet emerging as a shared determinant [1]. Early dietary intervention is gaining recognition as a modifiable strategy for reducing the incidence of psychiatric disorders [2]. The World Health Organization now acknowledges nutrition interventions as a fundamental approach in mental healthcare to address physical health disparities and the reduced life expectancy observed in individuals with certain psychiatric disorders [3].

A comprehensive strategy for both the prevention and treatment of psychiatric conditions must integrate lifestyle factors, including nutrition, but the provision of nutrition education to mental health providers is lacking [2]. Psychiatrists, psychologists, and psychotherapists may be unfamiliar with the growing body of evidence supporting dietary interventions in psychiatric care. For psychiatrists, this gap may stem from the broader deficiency of nutrition education in medical training [4]. Indeed, over two-thirds of psychiatrists and psychologists report having received no formal nutrition training [4]. While there is a pressing need to integrate nutrition education into psychiatric training, there is currently no established framework for doing so [5].

In this vein, a recent study demonstrated the feasibility and efficacy of an integrative psychiatry curriculum, which covered topics such as mind-body medicine, nutritional supplements, nutrition, and resident wellness [6]. However, to our knowledge, there are no curricula specific to nutrition management in psychiatric practice. Thus, the aim of this intervention was to develop a brief, structured curriculum to teach nutrition management in psychiatric practice to a general psychiatry residency program.

## Design and Development

The institutional review board determined that the assessment of this educational innovation was not human research (protocol #STU00219578). This curriculum was designed using a structured and evidence-based approach known as Kern’s six steps to curriculum design, as detailed below [7].

### Step 1: Problem Identification and General Needs Assessment

Based on our review of the literature described above, we determined that psychiatric residents receive inadequate training on nutritional assessment and interventions.

### Step 2: Needs Assessment of Targeted Learners

To address the problem of inadequate nutrition education in psychiatric training, we conducted a targeted needs assessment to identify key gaps in psychiatric education in our program. This comprised a self-administered, anonymous, voluntary, online survey which was sent to all psychiatry residents at McGaw Medical Center of Northwestern Memorial Hospital. The targeted needs assessment elicited subjective reports of knowledge base, subjective reports of skill set, preferences for specific content, and preferences for delivery format of the curriculum (i.e., flipped classroom, case-based learning, lectures, or online modules).

### Step 3: Goals and Objectives

Information gathered from the targeted needs assessment was used to create a brief structured curriculum on nutrition management in psychiatric clinical practice, as detailed in Table 1. The curriculum had three learning objectives: (1) affective change (i.e., increase confidence in engaging in nutrition management), (2) cognitive proficiency (i.e., improve clinical knowledge of nutrition management theory and resources), and (3) implementation (i.e., encourage use of this knowledge and skill set in a clinical scenario).

**Table 1:** Key themes and summary of content presented in didactic session, inclusive of material presented in initial curriculum and updated curriculum

**Key themes**	**Summary of content**
Highlight the importance of nutritional psychiatry	• Poor nutritional status is considered a modifiable risk factor for general mental health [2] • Mental health disorders are associated with poor nutritional status, including poor dietary choices, disordered eating behaviors, and nutritional deficiency [10] • Dietary interventions are accepted as a core strategy in mental healthcare to combat physical health inequalities and life-expectancy gap in individuals with psychiatric disorders [2, 3]
Review the mental health benefits of diet quality	• High diet quality (i.e., high intake of fruits, vegetables, fish, and whole grains) has been linked to improved well-being and improved outcomes in depressive disorders [11, 12] • Low diet quality (i.e., high intake of sugar and fat, low intake of fruits and vegetables) has been linked to higher risk of psychological distress, anxiety-like behaviors, and self-reported depression [13]
Review the mental health benefits of specific dietary patterns	• The MEDI-style diet has the most robust evidence for reducing risk of depression and anxiety [14–16] • Other dietary patterns, including DASH, MIND, and ketogenic diets, have theoretical benefits for mental health, but more evidence is needed [17, 18]
Review the mental health benefits and limitations of dietary supplements	• Nutraceuticals, or nutrient-based dietary supplements, and phytoceuticals, or plant-based dietary supplements, may be considered when there are barriers to implementing dietary patterns [5, 19] • The evidence supporting the use of supplements in psychiatric disorders was presented in accordance with previously published recommendations [5, 19] • Limitations to using dietary supplements include drug-supplement interactions, variability in supplement quality, limited data for use of supplements in specialized populations, and limited efficacy in severe mental illness [5]
Provide clinical resources for nutrition management	Patient-facing tools: • USDA MyPlate tool (to assess diet quality) • MEDI-style diet handout (to provide guidance on following MEDI-style diet) • USDA “Eating Healthy on a Budget” handout (to provide guidance on following a high quality diet on a budget) Provider-facing tools • Dietary supplements handout (to summarize evidence base for dietary supplements in psychiatric disorders) • NIH Nutrient Recommendations and Databases website (to provide additional information on supplement dosing and other considerations)

Abbreviations: *MEDI*, Mediterranean-style; *DASH*, Dietary Approaches to Stop Hypertension; *MIND*, Mediterranean-DASH Intervention for Neurodegenerative Delay; *USDA*, United States Department of Agriculture; *NIH*, National Institutes of Health

### Step 4: Educational Strategies

Based on these learning objectives, the educational strategy consisted of a two-session curriculum: a didactic session and a workshop session. The didactic session included (1) evaluation of participants’ knowledge of the theory and practice of nutrition management within a psychiatric clinical setting (i.e., a pre-assessment), (2) presentation of the evidence base and existing clinical resources for nutrition management within psychiatric practice as detailed in Table 1, and (3) assignment to conduct a nutrition assessment with a patient using knowledge and resources presented in the didactic session. Regarding the pre-assessment, there are no previously validated nutrition knowledge tests that have been implemented in this setting [8]. Therefore, a knowledge test was created based on material presented in the didactic session. The workshop session included (1) evaluation of participant’s knowledge of nutrition management within a psychiatric clinical setting (via post-assessment that mirrored the pre-assessment), (2) assessment of affective change (via anonymous feedback form which mirrored questions from the targeted needs assessment), (3) debrief on assignment to complete nutrition assessment in a clinical encounter (via open-ended questions which elicited self-report of resources used, successes, and challenges), and (4) acquisition of feedback on curriculum (via open-ended questions which probed helpful aspects of the curriculum, shortfalls of the curriculum, and ways in which the curriculum informed professional development of participating residents).

### Step 5: Implementation of the Curriculum

Of the 35 psychiatry residents enrolled in the program, 21 residents completed the targeted needs assessment. Subsequently, the initial curriculum was administered to seven (out of eight) residents in the post graduate year (PGY) 4 class. The didactic and workshop sessions were conducted across 1 h during existing protected time for didactics and administered approximately 1 month apart. Feedback on the initial curriculum was reviewed qualitatively and synthesized into key themes, without formal coding, to guide curriculum revision. The updated curriculum was administered to six (out of nine) residents in the PGY3 class during existing protected time for didactics. It followed the same two-session structure as the initial curriculum. Feedback gathered during the updated curriculum was reviewed qualitatively and synthesized into key themes, without formal coding, to inform future iterations of the curriculum. Residents were excluded from the curriculum if they were absent on the day that the didactic session was administered.

### Step 6: Evaluation

Efficacy of the intervention was collected as per three learning objectives. Affective change was measured qualitatively by comparing data from the targeted needs assessment with data from the anonymous feedback form administered in the workshop session. In the reporting of affective change, “yes” responses are inclusive of strongly agree and somewhat agree and “no” responses are inclusive of strongly disagree and somewhat disagree, due to small sample size. Cognitive proficiency was measured quantitatively by comparing performance on pre-assessment and post-assessment using two-tailed *t*-tests. Implementation of knowledge and skills in clinical encounters was assessed qualitatively through self-reported use of resources, without formal quantification. Due to the small sample size, results from the initial curriculum and updated curriculum were combined to assess affective change, cognitive proficiency, and implementation.

## Findings

### Targeted Needs Assessment

The targeted needs assessment demonstrated several key themes. Most of the participating residents were not confident in their ability to provide recommendations on a patient’s diet (76%, 16 out of 21) or provide recommendations on nutritional supplements (95%, 20 out of 21) as it relates to mental health. Most of the participating residents were unfamiliar or had not used existing clinical resources—i.e., United States Department of Agriculture MyPlate tool, National Institute of Health Nutrient Recommendations and Databases website for nutrition management (71–81%, 15 to 17 out of 21). Most of the participating residents did not feel that they had sufficient clinical knowledge (76%, 16 out of 21, somewhat disagree to strongly disagree) or sufficient clinical training (81%, 17 out of 21, somewhat disagree to strongly disagree) to incorporate nutrition management in their practice. Most of the participating residents were at least somewhat interested in learning more about nutrition assessment (95%, 20 out of 21). Lastly, most of the participating residents preferred more active learning formats, such as flipped classroom or case-based learning, rather than less interactive formats such as lectures or online modules (70%, 15 out of 21).

### Curriculum Delivery and Content

The topic of nutrition management is broad; therefore, the goals and limitations of the curriculum were explicitly described at the start of the didactic session. The goals of the curriculum are as listed in Table 1. Based on feedback from the targeted needs assessment, clinical cases were integrated within the didactic content to utilize residents’ preferred style of learning (i.e., case-based learning).

Feedback on the initial curriculum was reviewed qualitatively and synthesized into key themes, without formal coding, to guide curriculum revision. Residents identified the following as challenges to implementing the nutrition assessment with a patient: lack of confidence about their overall knowledge base of nutrition, lack of confidence about their skills to manage nutrition in a clinical setting, lack of patient-facing resources to be used outside of appointments, lack of knowledge about supplement dosing, lack of knowledge regarding supplement content, concerns about potentially dangerous combinations of supplements, and concerns about the cost of dietary interventions. When asked about missing resources, participants highlighted the need for alternative Mediterranean-style (MEDI) dietary patterns that are both affordable and culturally relevant, as well as sample daily meal plans to provide practical guidance. Additionally, participants requested independent study resources to further their learning outside of structured sessions.

The authors categorized feedback to determine whether suggested revisions should be incorporated into the existing two-part curriculum or reserved for a future advanced curriculum focused on more in-depth learning. Accordingly, the curriculum was revised to include resources on healthy eating on a budget and additional independent study materials, including online resources and peer-reviewed references. Electronic materials to disseminate clinical nutrition resources to patients could not be developed due to technical limitations within the electronic medical record system. Several topics were deemed more appropriate for an advanced curriculum and were not added to the updated curriculum. These included information on potentially harmful supplement combinations, culturally relevant adaptations of the MEDI diet, strategies for tailoring dietary recommendations to different patient populations, sample daily meal plans, and guidance on cost considerations and insurance coverage of supplements.

### Evaluation Metrics

Affective change was measured qualitatively by comparing pre-intervention targeted needs assessment data with workshop anonymous feedback data. Overall, participants reported improved comfort, clinical knowledge, and training in nutrition management. Confidence in providing dietary recommendations increased from 24% (5/21) pre-intervention to 77% (10/13) post-intervention, and confidence in recommending nutritional supplements rose from 5% (1/21) to 85% (11/13). Similarly, confidence in clinical knowledge increased from 5% (1/21) to 77% (10/13), and confidence in clinical training rose from 5% (1/21) to 69% (9/13).

Changes in cognitive proficiency were measured quantitatively using two-tailed *t*-tests to assess for statistically significant change in performance on the knowledge test before and after the didactic session. There was a statistically significant improvement between pre-intervention (mean = 5.23 ± 1.19) and post-intervention (mean = 7.62 ± 1.59) knowledge test (*t*(12) = −5.34, *p* < 0.001).

Lastly, participants discussed the clinical tools they utilized in patient encounters between the didactic and workshop sessions, highlighting several key themes. These included engaging in conversations about evidence-based dietary patterns, implementing the MyPlate assessment to evaluate nutritional intake, providing guidance on supplement use, and offering resources to address food insecurity. These discussions reflected the practical application of the curriculum content.

## Lessons Learned

To our knowledge, this is the first published description of a brief, structured curriculum to teach nutrition management in psychiatric practice to a general psychiatry residency program. The targeted needs assessment conducted before the intervention demonstrated that (1) most of the participating residents did not feel that they had sufficient knowledge or training in nutrition to engage in nutrition management within psychiatric clinical practice and (2) most of the participating residents were interested in learning more about nutrition management in psychiatric practice. This brief curriculum on nutrition management induced affective change, cognitive change, and implementation. In regard to affective change, residents indicated that they felt more confident engaging in nutrition management in psychiatric clinical practice after the intervention. Regarding cognitive change, residents demonstrated statistically significant improvement on a test of knowledge conducted before and after the didactic session. Lastly, residents were able to implement knowledge and resources introduced in the didactic session in a clinical encounter.

While the outcomes of this study are promising, the study had notable limitations. The sample size was very small, results from the initial curriculum and updated curriculum were combined due to small sample size, participants were at a single institution, there was no control group, and there was no longitudinal follow-up. Future studies with larger, multi-institutional samples, a control group, and longitudinal follow-up could validate these results, improve the generalizability of the findings, and assess sustained implementation of nutrition management in clinical practice. Building such a curriculum also comes with notable challenges, such as a lack of knowledgeable faculty. This barrier can be addressed by pairing a physician faculty member with a registered dietitian to share teaching responsibilities. This method has been effective in several primary care residency programs [9]. Additionally, this study was not designed to measure the extent to which the curriculum changed prescriber practices or patient outcomes. Future iterations might integrate electronic medical record data to capture these metrics. Lastly, there is a breadth of potentially relevant content for a nutritional psychiatry curriculum, and it is difficult to address all topics within a time-limited format. Future iterations of the curriculum could incorporate optional advanced sessions to explore specialized topics in greater depth.

In conclusion, nutrition is increasingly recognized as a core component of mental healthcare, yet medical education continues to fall short in preparing clinicians to manage nutrition in psychiatric practice. Given the growing burden of mental illness and the movement toward integrated models of care, there is a pressing need to expand nutrition education across all levels of psychiatric training. This study demonstrates the feasibility and preliminary effectiveness of a brief, structured curriculum on nutrition management within a psychiatry residency program. It can also serve as a framework from which other training programs can develop efficient and effective materials that support nutrition-focused care in psychiatric settings.
